# Noninvasive early detection of colorectal cancer by hypermethylation of the *LINC00473* promoter in plasma cell-free DNA

**DOI:** 10.1186/s13148-022-01302-x

**Published:** 2022-07-09

**Authors:** Juan Ruiz-Bañobre, Aitor Rodriguez-Casanova, Nicolas Costa-Fraga, Aida Bao-Caamano, Ana Alvarez-Castro, Martín Carreras-Presas, Elena Brozos-Vazquez, Yolanda Vidal-Insua, Francisca Vazquez-Rivera, Sonia Candamio-Folgar, Manuel Mosquera-Presedo, Ramón M. Lago-Lestón, Laura Muinelo-Romay, José Ángel Vázquez-Bueno, Rebeca Sanz-Pamplona, Víctor Moreno, Ajay Goel, Lourdes Castillo, Ana C. Martin, Rocio Arroyo, Manel Esteller, Ana B. Crujeiras, Rafael López-López, Angel Díaz-Lagares

**Affiliations:** 1 Translational Medical Oncology Group (ONCOMET), Health Research Institute of Santiago de Compostela (IDIS), University Clinical Hospital of Santiago (CHUS/SERGAS), 15706 Santiago de Compostela, Spain; 2 Centro de Investigación Biomédica en Red Cáncer (CIBERONC), ISCIII, 28029 Madrid, Spain; 3 Cancer Epigenomics Laboratory, Epigenomics Unit, Translational Medical Oncology Group (ONCOMET), Health Research Institute of Santiago de Compostela (IDIS), University Clinical Hospital of Santiago (CHUS/SERGAS), 15706 Santiago de Compostela, Spain; 4 Roche-Chus Joint Unit, Translational Medical Oncology Group (ONCOMET), Health Research Institute of Santiago (IDIS), 15706 Santiago de Compostela, Spain; 5 Universidade de Santiago de Compostela (USC), 15782 Santiago de Compostela, Spain; 6 Department of Gastroenterology and Hepatology, University Clinical Hospital of Santiago (CHUS/SERGAS), 15706 Santiago de Compostela, Spain; 7 Liquid Biopsy Analysis Unit, Translational Medical Oncology Group (ONCOMET), Health Research Institute of Santiago de Compostela (IDIS), 15706 Santiago de Compostela, Spain; 8 Department of Pathology, Complejo Hospitalario Universitario de Ferrol (SERGAS), 15405 Ferrol, Spain; 9 Unit of Biomarkers and Susceptibility, Oncology Data Analytics Program, Catalan Institute of Oncology (ICO), 08907 Barcelona, Spain; 10 Colorectal Cancer Group, Bellvitge Biomedical Research Institute (IDIBELL), 08907 Barcelona, Spain; 11 Biomedical Research Centre Network for Epidemiology and Public Health (CIBERESP), 28029 Madrid, Spain; 12 Department of Clinical Sciences, Faculty of Medicine and Health Sciences, University of Barcelona, 08907 Barcelona, Spain; 13 Center for Gastrointestinal Research, Center for Translational Genomics and Oncology, Baylor Scott and White Research Institute, Charles A Sammons Cancer Center, Baylor University Medical Center, Dallas, TX USA; 14 Department of Molecular Diagnostics and Experimental Therapeutics, Beckman Research Institute of City of Hope, Monrovia, CA USA; 15 City of Hope Comprehensive Cancer Center, Duarte, CA USA; 16 Advanced Marker Discovery (AMADIX), 47004 Valladolid, Spain; 17 Josep Carreras Leukaemia Research Institute (IJC), Barcelona, Spain; 18 Institucio Catalana de Recerca I Estudis Avançats (ICREA), Barcelona, Spain; 19 Physiological Sciences Department, School of Medicine and Health Sciences, University of Barcelona (UB), Barcelona, Spain; 20 Epigenomics in Endocrinology and Nutrition Group, Epigenomics Unit, Health Research Institute of Santiago de Compostela (IDIS), University Clinical Hospital of Santiago (CHUS/SERGAS), 15706 Santiago de Compostela, Spain; 21 Centro de Investigación Biomédica en Red Fisiopatología de La Obesidad y Nutrición (CIBERobn), ISCIII, 28029 Madrid, Spain

**Keywords:** DNA methylation, *LINC00473*, Cell-free DNA, Early detection, Colorectal cancer

## Abstract

**Background:**

Current noninvasive assays have limitations in the early detection of colorectal cancer. We evaluated the clinical utility of promoter methylation of the long noncoding RNA *LINC00473* as a noninvasive biomarker to detect colorectal cancer and associated precancerous lesions.

**Methods:**

We evaluated the epigenetic regulation of *LINC00473* through promoter hypermethylation in colorectal cancer cell lines using bisulfite genomic sequencing and expression analyses. DNA methylation of *LINC00473* was analyzed in primary colorectal tumors using 450K arrays and RNA-seq from The Cancer Genome Atlas (TCGA). Tissue-based findings were validated in several independent cohorts of colorectal cancer and advanced colorectal polyp patients by pyrosequencing. We explored the clinical utility of *LINC00473* methylation for the early detection of colorectal cancer in plasma cell-free DNA by quantitative methylation-specific PCR and droplet digital PCR.

**Results:**

*LINC00473* showed transcriptionally silencing due to promoter hypermethylation in colorectal cancer cell lines and primary tumors. Methylation of the *LINC00473* promoter accurately detected primary colorectal tumors in two independent clinical cohorts, with areas under the receiver operating characteristic curves (AUCs) of 0.94 and 0.89. This biomarker also identified advanced colorectal polyps from two other tissue-based clinical cohorts with high diagnostic accuracy (AUCs of 0.99 and 0.78). Finally, methylation analysis of the *LINC00473* promoter in plasma cell-free DNA accurately identified patients with colorectal cancer and advanced colorectal polyps (AUCs of 0.88 and 0.84, respectively), which was confirmed in an independent cohort of patients.

**Conclusions:**

Hypermethylation of the *LINC00473* promoter is a new promising biomarker for noninvasive early detection of colorectal cancer and related precancerous lesions.

**Supplementary Information:**

The online version contains supplementary material available at 10.1186/s13148-022-01302-x.

## Background

Colorectal cancer (CRC) is the third most frequently detected cancer in both sexes worldwide and is expected to increase by 60% to more than 2.2 million new cases by 2030 [1]. CRC is usually diagnosed at an advanced stage of disease [2] and represents a leading cause of cancer mortality worldwide [3]. For CRC patients, the 5‐year survival rate ranges from 90 to 14% depending on whether they are diagnosed at early or advanced stages, respectively [4]. The growing incidence and high mortality rates of CRC highlight the clinical need for novel strategies to improve early CRC detection and patient management [5].

Colorectal carcinogenesis is a multistep process involving genetic and epigenetic alterations [6]. The majority of CRCs (70%) originate from a common colorectal precursor lesion, adenomatous polyp or conventional adenomas, which can potentially become malignant through the “traditional” carcinogenesis pathway (adenoma–carcinoma sequence). In addition, another type of colorectal lesion, serrated polyps, has been recently recognized as a precursor of 30% of all CRCs through the “serrated” carcinogenesis pathway. Among these precancerous lesions, some are defined as advanced colorectal polyps (ACPs) and present a higher risk of cancer transformation [7]. There are several screening assays used to detect CRC at an early stage, including the fecal immunochemical test (FIT), which is a widely used noninvasive and cost-effective assay for detecting the presence of fecal hemoglobin [8]. However, this type of stool-based assay has shown some limitations, including the inability to reliably detect colorectal precancerous lesions, such as ACPs [9]. Although colonoscopy is considered the gold standard CRC diagnosis technique, it is an invasive procedure that, in addition to requiring tedious and time-consuming preparation, can potentially cause serious complications [10] and has low patient adherence [11]. Therefore, novel non‐invasive tests for early detection of CRC are urgently needed.

Long noncoding RNAs (lncRNAs) are an emerging group of heterogeneous noncoding transcripts longer than 200 nt involved in the regulation of many biological processes in normal cells [12]. Of note, the expression of lncRNAs can be disrupted in cancer by several mechanisms, such as hypermethylation of CpG islands (CpGIs) in their promoters [13, 14]. DNA methylation is a well-known epigenetic mechanism based on the incorporation of a methyl group (CH_3_) into the 5′ carbon of cytosines in cytosine-phosphate-guanine (CpG) dinucleotides that generates 5-methylcytosine (5mC). In carcinogenesis, hypermethylation of tumor suppressor genes (TSGs) represents an early event usually associated with their transcriptional silencing, which leads to tumor initiation and disease progression. This epigenetic mechanism can be detected in tumors but also in cell-free DNA (cfDNA) released into circulation by tumor cells, showing great potential as a dynamic and noninvasive tool for CRC diagnosis [5].

*LINC00473* is a lncRNA downregulated in CRC that exerts tumor suppressor functions in this disease by promoting apoptotic protease-activating factor 1 (APAF1) IRES activity through competitively sponging miR574-5p and miR15b-5p in tumor initiation and pathogenesis [15]. Of note, Diaz-Lagares et al. recently identified that the CpGI in the promoter of *LINC00473* is hypermethylated in CRC [14]. Therefore, in this study, we evaluated the epigenetic regulation of *LINC00473* by DNA methylation and its clinical impact for noninvasive early detection of CRC.

## Results

### Epigenetic regulation of *LINC00473* by promoter methylation in colorectal cancer

**Fig. 1:**
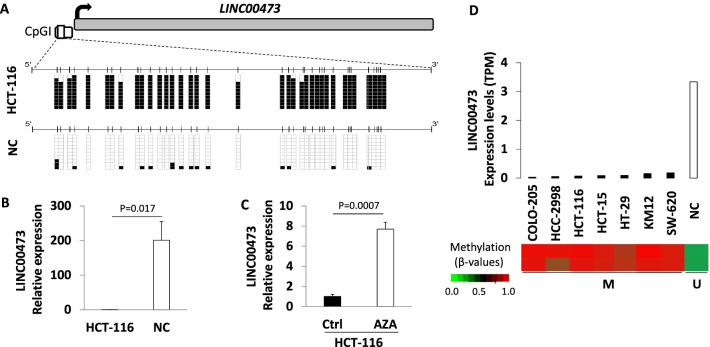
Epigenetic silencing of the *LINC00473* in colorectal cancer cells. **A** Bisulfite genomic sequencing analysis of *LINC00473* promoter CpG island in the colorectal cancer cell line HCT-116 and normal tissue. Locations of CpG dinucleotides (vertical lines) and the TSS (long black arrow) are shown. Ten single clones are represented for each sample. The presence of unmethylated and methylated CpGs is indicated by white and black squares, respectively. **B** DNA methylation-associated transcriptional silencing of *LINC00473* in the colorectal cancer cell line HCT-116. Expression levels of *LINC00473* were determined by qRT-PCR in the methylated cancer cell line HCT-116 and in colorectal normal tissues (N = 3). **C** Restored *LINC00473* expression in the methylated cancer cell line HCT-116 after AZA treatment analyzed by qRT-PCR. Values were determined from triplicates and are expressed as the mean ± SEM. **D** Methylation and expression analysis of *LINC00473* in common colorectal cancer cell lines and normal colon mucosa using 450K array and RNA-seq public datasets. Expression levels obtained by RNA-seq were expressed as transcripts per million (TPMs). TSS, transcription start site; NC; normal colon mucosa

CpGI hypermethylation in the promoter of *LINC00473* has been recently described in the colorectal cancer cell line HCT-116 using the Infinium HumanMethylation450 (450K) microarray (Illumina) [14]. To confirm this epigenetic feature, we first analyzed the methylation status of *LINC00473* in HCT-116 cells in comparison with normal colon mucosa by bisulfite genomic sequencing of multiple clones (Fig. 1A). Importantly, the CpGI promoter region of *LINC00473* that we analyzed contains CpGs included in the 450K array. As expected, we observed hypermethylation of all the CpGs analyzed in HCT-116 cells compared to unmethylated normal colon mucosa. The effect of the CpGI hypermethylation on gene expression (Fig. 1B) was analyzed by qRT-PCR, showing the downregulation of *LINC00473* in HCT-116 cells with respect to unmethylated normal colon mucosa. Importantly, the use of the demethylating agent 5-aza-2’-deoxycytidine (AZA) in HCT-116 cells restored the expression of *LINC00473* (Fig. 1B, C). Thus, we extended the methylation and expression analysis to other common colorectal cancer cell lines (Fig. 1D) using 450K array and RNA-seq datasets obtained from Gene Expression Omnibus (GEO): GSE49143 and GSE138734, respectively. These analyses confirmed CGI promoter hypermethylation and downregulation of *LINC00473* in all CRC cell lines analyzed (COLO-205, HCC-2998, HCT-116, HCT-15, HT-29, KM12, and SW-620) in comparison with normal colon mucosa.

### Methylation status of the *LINC00473* promoter in colorectal tumors

**Fig. 2:**
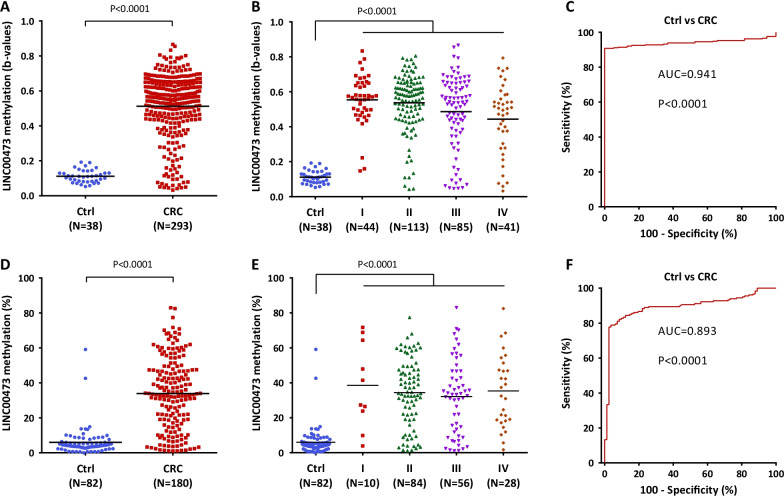
Methylation of *LINC00473* in colorectal cancer tissues. Methylation analysis of *LINC00473* promoter in tissues from primary CRC and matched normal colorectal mucosa (controls) of two independent cohorts analyzed by 450K array (Cohort 1) and pyrosequencing (Cohort 2). **A**, **B** Methylation status of *LINC00473* in (**A**) all CRC patients (N = 293) and (**B**) in those CRC patients (N = 283) with available clinical information on their TNM tumor stage (Cohort 1). **C** ROC curve analysis evaluating the methylation of *LINC00473* for the detection of CRC in tissue samples in Cohort 1. **D**, **E** Validation of the methylation status of *LINC00473* in (**A**) all CRC patients (N = 180) and (**B**) in those CRC patients (N = 178) with available clinical information on their TNM tumor stage in Cohort 2. **F** ROC curve analysis to validate the methylation of *LINC00473* for the detection of colorectal tumors in Cohort 2. Horizontal lines represent mean methylation levels of *LINC00473*. P, *p* value analyzed by Mann–Whitney *U* test or ROC curve; AUC, area under the ROC curve; Ctrl, controls; CRC, colorectal cancer

After confirming the epigenetic regulation of *LINC00473* by promoter methylation in CRC cell lines, we decided to evaluate whether this epigenetic alteration was a general event in primary colorectal tumors. Thus, we evaluated the methylation status of the *LINC00473* promoter and the expression level of the corresponding gene by using a 450K array and RNA-seq, respectively, from a TCGA dataset (Cohort 1) of primary colorectal tumors (from TNM stage I to IV) and matched normal tissues (controls). As expected from our results with CRC cell lines, this analysis revealed a significantly higher methylation level of *LINC00473* in colorectal tumors than in controls (Fig. 2A), which was consistent across all TNM tumor stages (Fig. 2B). In addition, *LINC00473* promoter hypermethylation was associated with a significant downregulation of its expression levels in primary colorectal tumors (Additional file 1: Fig. S1). Consistent with this, promoter methylation of *LINC00473* showed a significant inverse correlation (*r* = − 0.19; *p* = 0.0009) with its expression levels in CRC patients from the TCGA dataset (Cohort 1).

Next, we generated receiver operating characteristic curves (ROCs) to evaluate the robustness of the methylation status of the *LINC00473* promoter for CRC diagnosis across all tumor stages. This analysis revealed a significantly high CRC detection accuracy with an area under the receiver operating characteristic curve (AUC) of 0.941 (95% CI 0.915–0.966, *p* < 0.0001) (Fig. 2C), a sensitivity of 91% (CI 95% 87–94%), a specificity of 100% (CI 95% 91–100%), a positive predictive value (PPV) of 100% and a negative predictive value (NPV) of 58%. Moreover, ROC curve analyses yielded a very high diagnostic accuracy across all tumor stages separately (Additional file 2: Fig. S2). Subsequently, these results were confirmed in an independent cohort (Cohort 2) of primary colorectal tumors (from TNM stage I to IV) and matched non-tumor controls using bisulfite pyrosequencing (Fig. 2D–F). This analysis also showed a significantly higher methylation level of the *LINC00473* promoter in primary colorectal tumors than in controls (Fig. 2D), which was constant across all TNM tumor stages (Fig. 2E). As expected, the *LINC00473* methylation level identified primary colorectal tumors with an AUC of 0.893 (95% CI 0.851–0.935, < 0.0001) (Fig. 2F), a sensitivity of 78% (CI 95% 71–84%), a specificity of 98% (CI 95% 92–100%), a PPV of 99% and an NPV of 67%. The detection accuracy was constant across all CRC stages separately, as shown in Additional file 3: Fig. S3.

### Methylation status of the *LINC00473* promoter in tissue from precancerous colorectal lesions

**Fig. 3:**
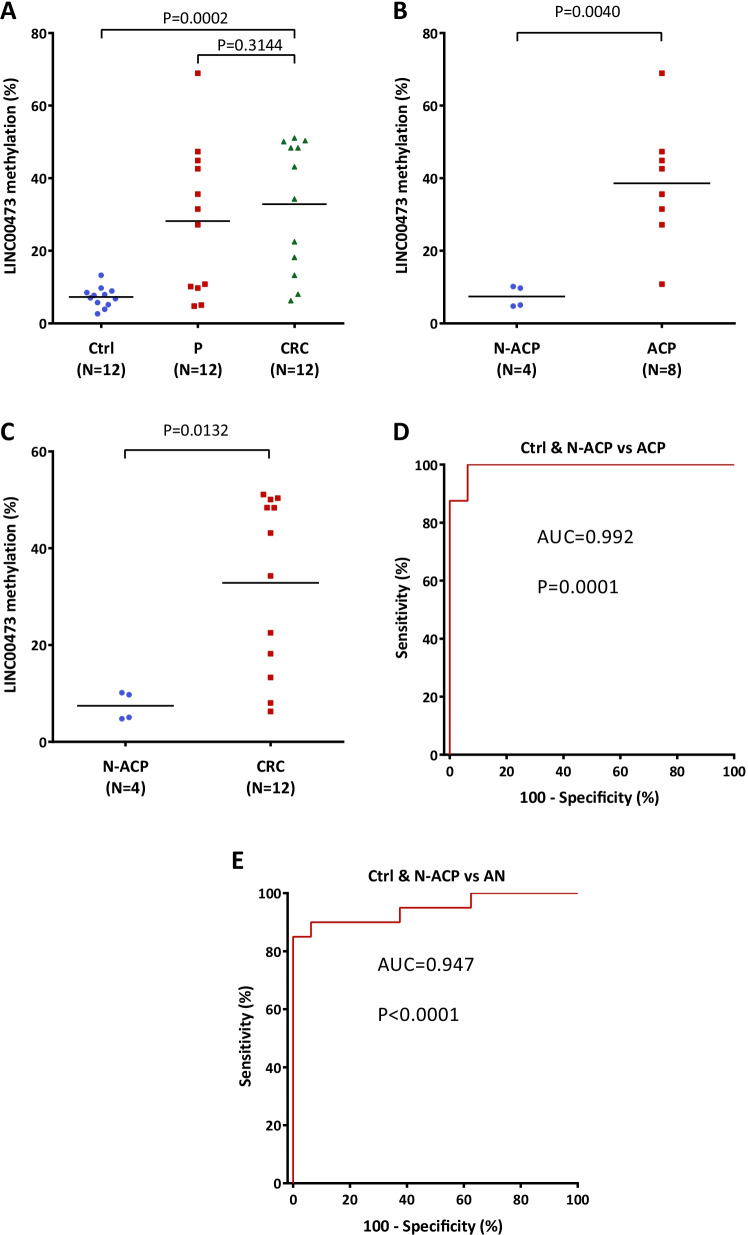
Methylation levels of *LINC00473* in tissue precancerous colorectal lesions. **A** Methylation levels of *LINC00473* promoter in tissues from premalignant colorectal polyps, CRC and matched normal colorectal mucosa (controls) by pyrosequencing (Cohort 3). **B**, **C** Methylation levels of *LINC00473* promoter in tissues from N-ACP, ACP and CRC (Cohort 3) analyzed by pyrosequencing. **D** ROC curve analysis evaluating the methylation of *LINC00473* promoter for the detection of ACPs with respect to the combination of controls and N-ACPs (Cohort 3). **E** ROC curve to evaluate the methylation of *LINC00473* promoter for the detection of AN with respect to the combination of controls and N-ACPs (Cohort 3). Horizontal lines represent mean methylation levels of *LINC00473*. P, *p* value analyzed by Mann–Whitney *U* test or ROC curve; AUC, area under the ROC curve; Ctrl, controls; P, polyps; CRC, colorectal cancer; N-ACP, non-advanced colorectal polyps; ACP, advanced colorectal polyps; AN, advanced neoplasia

DNA methylation is an epigenetic mechanism that can be deregulated in precancerous colorectal lesions [16]. To confirm this feature, we analyzed the methylation status of the *LINC00473* promoter by bisulfite pyrosequencing in tissues from premalignant colorectal polyps, CRC and matched normal colorectal mucosa (controls) (Cohort 3) (Fig. 3). As expected, the methylation of *LINC00473* was significantly higher in polyps and in CRC than in healthy controls, while no differences were found between polyps and CRC (Fig. 3A). Importantly, from a clinical viewpoint, we also found significant differences between *LINC00473* methylation levels in non-ACP (N-ACP) compared to ACP (Fig. 3B) and in N-ACP compared to CRC (Fig. 3C) but not in controls compared to N-ACP or in ACP compared to CRC (Additional file 4: Fig. S4A-B). Next, we generated ROC curves to evaluate the robustness of this methylation biomarker to detect CRC or premalignant colorectal polyps, and we observed a high capacity of *LINC00473* to differentiate controls from polyps (AUC = 0.840, CI 95% 0.657–1.00, *p* = 0.0047; sensitivity = 83%, CI 95% 52–98%; specificity = 92%, CI 95% 62–100%; PPV = 91%; NPV = 85%) and controls from CRC (AUC = 0.917, CI 95% 0.794–1.00, *p* = 0.0005; sensitivity = 83%, CI 95% 52–98%; specificity = 100%, CI 95% 74–100%; PPV = 100%; NPV = 86%) (Additional file 4: Fig. S4C-D). More importantly, the methylation status of *LINC00473* was able to accurately detect ACP (AUC = 0.992, CI 95% 0.967–1, *p* = 0.0001; sensitivity = 100%, CI 95% 63–100%; specificity = 94%, CI 95% 70–100%, PPV = 89%; NPV = 100%) (Fig. 3D) and AN (AUC = 0.947, CI 95% 0.873–1.00, *p* < 0.0001; sensitivity = 85%, CI 95% 62–97%; specificity = 100%, CI 95% 79–100%, PPV = 100%; NPV = 84%) (Fig. 3E).

To better appreciate the translational potential of our previous findings, we asked whether this methylation biomarker could be successfully validated using pyrosequencing in an additional cohort (cohort 4) composed of colorectal polyps and normal mucosa from non-cancer patients (controls). In addition to confirming methylation differences between controls and polyps (Additional file 5: Fig. S5A), with a larger number of N-ACPs, in this cohort, we were able to detect significantly higher methylation levels in controls than in N-ACPs (Additional file 5: Fig. S5B). Importantly from a clinical viewpoint, we confirmed significantly higher methylation levels of *LINC00473* in ACPs than in N-ACPs (Additional file 5: Fig. S5C). Furthermore, ROC curves demonstrated the high accuracy of the methylation status of *LINC00473* to detect polyps (AUC = 0.831, CI 95% 0.738–0923, *p* = 0.0009; sensitivity = 70%, CI 95% 57–81%; specificity = 100%, CI 95% 69–100%, PPV = 100%; NPV = 36%) (Additional file 5: Fig. S5D) and, even more importantly, to detect ACPs (AUC = 0.776, CI 95% 0.665–0.888, *p* < 0.0001; sensitivity = 71%, CI 95% 54–85%; specificity = 79%, CI 95% 62–91%, PPV = 78%; NPV = 73%) (Additional file 5: Fig. S5E).

Additionally, we also assayed for a possible effect of age on *LINC00473* methylation levels in tissue samples; however, no significant effect was found (*p* > 0.05). Furthermore, we also did not find any significant difference (*p* > 0.05) in methylation levels according to the sex of the individuals (data not shown).

### Diagnostic potential of methylation of the *LINC00473* promoter to detect colorectal cancer and precancerous lesions in plasma cell-free DNA

**Fig. 4:**
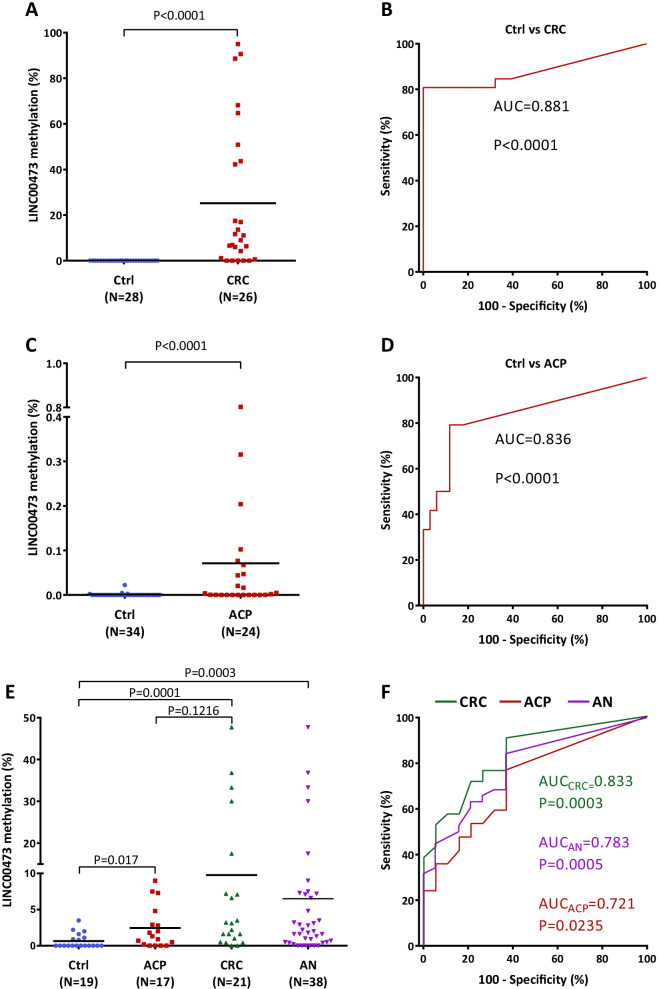
Methylation of *LINC00473* in cfDNA from patients with advanced colorectal polyps and colorectal cancer. **A** Methylation status of *LINC00473* promoter in plasma cfDNA of CRC patients analyzed by qMSP (Cohort 5). **B** ROC curve analysis evaluating the methylation of *LINC00473* for the detection of CRC in plasma cfDNA (Cohort 5). **C**, Methylation status of *LINC00473* promoter in plasma cfDNA of patients with ACP analyzed by qMSP (Cohort 6). **D** ROC curve analysis evaluating the methylation of *LINC00473* for the detection of ACP in plasma cfDNA (Cohort 6). **E** Validation of the methylation status of *LINC00473* promoter in plasma cfDNA of patients with ACP by ddPCR analysis (Cohort 7). **F**, ROC curve analysis to validate the methylation of *LINC00473* for the detection of ACP and CRC in plasma cfDNA (Cohort 7). Horizontal lines represent mean methylation levels of *LINC00473*. P, *p* value analyzed by Mann–Whitney *U* test or ROC curve; AUC, area under the ROC curve; Ctrl, controls. ACP, advanced colorectal polyps; CRC, colorectal cancer.

Beyond tissue samples, promoter hypermethylation of several genes in plasma cfDNA of patients with ACPs or CRC has also been described [5]. Based on this fact, we analyzed the methylation of the *LINC00473* promoter in plasma cfDNA of a cohort of self-declared healthy controls and CRC patients by qMSP (Cohort 5). The result of this analysis showed significantly higher methylation levels in CRC than in controls (Fig. 4A). In addition, the methylation levels of *LINC00473* in cfDNA exhibited a very high diagnostic accuracy to detect CRC patients with an AUC of 0.881 (CI 95% 0.776–0983, *p* < 0.0001), a sensitivity of 81% (95% CI 61–93%), a specificity of 100% (95% CI 88–100%), a PPV of 100% and an NPV of 85% (Fig. 4B). Furthermore, we also analyzed the plasma cfDNA of a cohort of self-declared healthy controls and patients with ACPs presenting at least one polyp > 10 mm previously confirmed by colonoscopy (Cohort 6). Of note, this assay revealed significantly higher methylation levels of *LINC00473* in cfDNA of ACPs than in controls (Fig. 4C), showing a high diagnostic accuracy to detect ACPs (AUC = 0.836; 95% CI 0.722–0.949, *p* < 0.0001) with a sensitivity of 79% (95% CI 58–93%), a specificity of 88% (95% CI 73–97%), a PPV of 83% and an NPV of 86% (Fig. 4D).

To confirm the feasibility of using methylation of the *LINC00473* promoter for the noninvasive early detection of CRC, we retrospectively analyzed an independent cohort of plasma cfDNA samples (Cohort 7) obtained either prior to a scheduled colonoscopy as part of standard CRC screening or prior to colonic surgery for primary tumors (Fig. 4E, F). Due to the need for very sensitive methodologies for the early detection of cancer in liquid biopsy [5], we used ultrasensitive droplet digital PCR (ddPCR) for the methylation analysis of *LINC00473*. Consistent with our previous results in tissue and cfDNA, the methylation levels of *LINC00473* were significantly higher in ACP and CRC than in confirmed healthy controls (Fig. 4E). In addition, no significant differences were found between the methylation status of *LINC00473* of ACP and CRC (Fig. 4E). Of note, methylation of the *LINC00473* promoter showed high accuracy for the detection of ACP, with an AUC of 0.721 (95% CI 0.553–0.890, *p* = 0.0235), a sensitivity of 76% (95% CI 50–93%), a specificity of 63% (95% CI 38–84%), a PPV of 65% and an NPV of 75% (Fig. 4F). Similarly, methylation of *LINC00473* was able to identify CRC patients with a high AUC of 0.833 (95% CI 0.709–0.958, *p* = 0.0003), a sensitivity of 90% (95% CI 70–99%), a specificity of 63% (95% CI 38–84%), a PPV of 73 and an NPV of 86. In addition, *LINC00473* showed the ability to detect AN with an AUC of 0.783 (95% CI 0.662–0.904, *p* = 0.0005), a sensitivity of 84% (95% CI 69–94%), a specificity of 63% (95% CI 38–84%), a PPV of 82% and an NPV of 67% (Fig. 4F).

Similar to our previous analysis in tissue samples, we also found no significant effect of age (*p* > 0.05) on *LINC00473* methylation levels in cfDNA samples. In addition, no significant difference (*p* > 0.05) in methylation levels according to the sex of the individuals was found (data not shown).

### Clinical utility of *LINC00473* promoter methylation for the noninvasive detection of colorectal cancer during the follow-up of metastatic patients

After confirming its utility in the detection of precancerous lesions and CRC, we decided to evaluate whether the methylation status of the *LINC00473* promoter in plasma cfDNA could be useful to diagnose the presence of CRC in the palliative setting. For this purpose, we analyzed the methylation of the *LINC00473* promoter in plasma cfDNA by ddPCR at various clinically relevant time points from a cohort of six randomly selected mCRC patients (Cohort 8), whose disease evolution was evaluated according to a standard clinical practice (serial serum carcinoembryonic antigen (CEA) determinations and computed tomography scans). As shown in Additional file 6: Fig. S6, the plasma cfDNA methylation levels of *LINC00473* decreased with effective therapy and increased with disease progression. Notably, in some cases (e.g., cases #1, #4, and #5), *LINC00473* methylation preceded CEA in detecting the presence of CRC. Together, these six cases represent proof of concept of the utility of *LINC00473* methylation as a potential biomarker to detect the presence of CRC during the follow-up of metastatic patients.

## Discussion

Colorectal cancer (CRC) is one of the most common malignancies and is a major cause of cancer-related deaths worldwide [17]. The high incidence and mortality of CRC highlight the clinical need for novel strategies to improve early detection and personalize the management of patients with this type of tumor. Deregulation of epigenetic mechanisms, such as the promoter hypermethylation of lncRNAs, has relevant implications for CRC development and progression [14, 18]. Importantly, DNA methylation can be detected both in tumor cells and in various components of liquid biopsy, such as cfDNA, and has shown clinical utility as a cancer biomarker [5]. Thus, in this work, we studied epigenetic regulation through DNA methylation of lncRNA *LINC00473* and evaluated the clinical utility of hypermethylation of its promoter for the detection of CRC and its precancerous lesions in both tissue and noninvasively collected patient samples.

*LINC00473* is a lncRNA with pro-apoptotic tumor suppressor properties in CRC whose expression is downregulated in this tumor type. This lncRNA is able to sponge endogenous miR574-5p or miR15b-5p, inhibit cell proliferation and colony formation capacity, and induce cell apoptosis by activating the APAF1-CASP9-CASP3 pathway [15]. In addition, a genome-wide analysis of the CRC cell line HCT-116 recently identified promoter hypermethylation of *LINC00473* [14]. However, information is lacking on the methylation status of *LINC00473* in CRC and the regulation of this lncRNA by this epigenetic mechanism. Thus, in this work, we confirmed that *LINC00473* is hypermethylated in CRC and that this epigenetic modification is associated with the transcriptional silencing of this lncRNA. In line with this, previous works have described the aberrant hypermethylation of various lncRNAs in several tumor types, including CRC [14, 19].

The deregulation of DNA methylation is an epigenetic alteration that takes place during colorectal carcinogenesis and can be detected from precancerous lesions, such as ACP, to advanced stages of CRC [5]. In this context, our study revealed that the promoter of *LINC00473* is hypermethylated in primary colorectal tumors and that this epigenetic deregulation is present from early to advanced stages of CRC. Importantly, *LINC00473* was also hypermethylated in ACP tissues, indicating that the hypermethylation of *LINC00473* is an early event in colorectal carcinogenesis that is present throughout all stages of tumor development. In addition, we found in primary colorectal tumors that the hypermethylation of *LINC00473* was associated with a decrease in the expression of this lncRNA. These results are in line with a previous work, which identified the downregulation of expression and tumor suppressor properties of *LINC00473* in association with the initiation and pathogenesis of CRC [15]. Similarly, other authors have found epigenetic deregulation of important TSGs, such as *p16/CDKN2A* and *hMLH1*, throughout the colorectal carcinogenesis process [20, 21]. In addition to biological effects on gene expression, epigenetic alterations have shown clinical utility as tumor biomarkers at different stages of CRC disease [5]. In this regard, we found that the methylation status of *LINC00473* in tissue samples was able to accurately identify ACPs and the different stages of CRC from early to advanced disease, indicating the feasibility of using the methylation of *LINC00473* as a biomarker for the early detection of CRC.

There is a pressing need in the clinic to detect CRC at the beginning of the disease, since the majority of patients can be successfully treated if detected early [4]. Although colonoscopy is an effective diagnostic tool for the screening and early detection of CRC, it is an invasive method that limits its application [10]. Currently, FIT is a noninvasive stool-based assay widely used for the early detection of CRC in screening programs; however, it has a low sensitivity for the early detection of precancerous lesions [9]. To date, several noninvasive biomarkers have been proposed for the early detection of CRC, including circulating epigenetic biomarkers [9]. Alterations in DNA methylation are promising candidates for early diagnosis, since they are covalent and stable marks that can be found early in carcinogenesis [22]. In line with this, the detection of *SEPT9* methylation in plasma cfDNA (Epi proColon), which was approved by the US Food and Drug Administration (FDA) in 2016 and has been proposed as a noninvasive test for the early detection of CRC [23]. However, a large prospective study revealed that the detection of *SEPT9* methylation has low diagnostic accuracy for CRC (sensitivity, 48.2%) and advanced precancerous lesions (sensitivity, 11.2%) [24], highlighting the need for new accurate, noninvasive tests for the early detection and management of CRC. In this work, we found that, similar to tissue samples, the promoter of *LINC00473* was hypermethylated in plasma cfDNA of CRC and ACP patients. Of note, the methylation of *LINC00473* in cfDNA allowed the identification of CRC and ACP with very good AUCs, indicating that it is an assay with high diagnostic accuracy for the very early detection of CRC [25]. In addition to showing interesting specificity and PPV, methylation of the *LINC00473* promoter in cfDNA also showed high sensitivity and NPV in a CRC and precancerous lesions screening context. While high PPV is desired when the costs or risks of further testing are significant, NPV gains importance when the disease is serious and curable in its preclinical phase, as is the case of ACP and CRC [26]. Another advantage of analyzing the methylation of *LINC00473* in plasma would be the higher adherence that blood-based assays provide in comparison with widely used fecal tests, such as FIT [27].

While we only performed a small proof-of-concept study, the methylation levels *of LINC00473* in cfDNA were able to detect the presence of the disease during the follow-up of metastatic CRC patients, anticipating, in some cases, the response and progression compared with a standard protocol based on serial CEA determinations and CT scans [28]. These results show the promising potential of *LINC00473* methylation in cfDNA to facilitate disease detection in the palliative setting of CRC patients.

Altogether, the results of this work indicate that the methylation status of *LINC00473* in cfDNA is a promising biomarker for the noninvasive early detection of CRC and its precancerous lesions that could be used in the clinic. Although methylation analysis of cfDNA was performed in a limited number of patients, our results support further validation to confirm the clinical applications of this novel epigenetic biomarker in large-scale prospective studies with asymptomatic screening participants and colorectal cancer patients. Because the combination of different types of biomarkers may increase the accuracy of diagnostic assays [22], future studies should also evaluate whether the combination of *LINC00473* with other circulating biomarkers may improve its high diagnostic accuracy for the early detection of CRC.

## Conclusions

CRC is a leading cause of cancer mortality worldwide that is usually diagnosed at an advanced stage, highlighting the need for new early detection strategies. We evaluated the clinical utility of the promoter methylation of the long noncoding RNA *LINC00473* in plasma cfDNA as a noninvasive biomarker for the detection of CRC. The methylation of *LINC00473* showed high diagnostic accuracy to detect CRC and associated precancerous lesions both in tissues and in cfDNA, indicating that the methylation of *LINC00473* has a huge potential as a biomarker for the noninvasive early detection of CRC and related precancerous lesions.

## Methods

### Cancer cell lines and treatments

The human CRC cell line HCT-116 (American Type Culture Collection [ATCC]) was cultured in DMEM with GlutaMAX (Gibco) supplemented with 10% fetal bovine serum (Sigma-Aldrich) and 1% penicillin/streptomycin (Gibco) at 37 °C and 5% CO_2_. To promote DNA demethylation, HCT-116 cells were treated with 5-aza-2′-deoxycytidine (AZA) (Sigma-Aldrich) at 5 μM for 72 h. Treatment was performed in triplicate, and data were compared with the corresponding non-treated cell line.

### Study participants

In this retrospective study, methylation levels of *LINC00473* were analyzed in eight independent clinical cohorts of patients, including four cohorts of colorectal tissue samples (Cohorts 1 to 4) and four cohorts of plasma samples (Cohorts 5 to 8) (Additional file 7: Table S1). Polyps were categorized into advanced colorectal polyps (ACPs) and non-ACPs (N-ACPs). ACP was defined as adenomas of at least 10 mm in size, containing high-grade dysplasia or with tubulovillous or villous histology, or a serrated polyp of at least 10 mm in size or containing any grade of dysplasia. Advanced neoplasia (AN) was defined as CRC or ACP [29]. The main clinical characteristics of the respective cohorts are described in Additional file 8: Table S2 and Additional file 9: Table S3. Inclusion and exclusion criteria are detailed in Additional file 10: Methods.

### Blood sample collection and plasma isolation

Blood samples were collected by phlebotomy into collection tubes with EDTA as an anticoagulant. Plasma was isolated within 2 h after collection by an initial centrifugation at 1,500–1,600 g for 10 min at 4 °C, followed by a second centrifugation at 15,000–16,000 g for 10 min at 4 °C. Isolated plasma was stored at −80 °C until analysis.

### Isolation of nucleic acids from cell lines, tissues and plasma samples

Genomic DNA (gDNA) and total RNA were isolated from cell lines using TRIzol (Invitrogen) according to the manufacturer's protocol. gDNA was also isolated from formalin-fixed paraffin-embedded (FFPE) colorectal tissues with the AllPrep DNA/RNA FFPE Kit (Qiagen). cfDNA was isolated from 2–4 mL of plasma using the QIAamp® Circulating Nucleic Acid Kit (Qiagen) and the vacuum system QIAvac 24 Plus (Qiagen) following the manufacturer's recommendations. The quality and quantity of gDNA and RNA were evaluated with a NanoDrop (Thermo Fisher), and cfDNA was quantified by the QuantiFluor® ONE dsDNA kit with the Quantus™ Fluorometer (Promega). DNA and RNA were stored at − 80 °C until analysis.

### Bisulfite genomic sequencing

Methyl Primer Express v1.0 software (Applied Biosystems) and Primer3 (v.0.4.0) were used to design primers for the methylation analysis. DNA (1 µg) was subjected to sodium bisulfite treatment using the EZ DNA Methylation-Gold kit (Zymo Research). A 490-bp fragment of the *LINC00473* promoter was amplified using 2 μL of bisulfite-converted DNA with ImmolaseTaq polymerase (Bioline) at 60 °C for 40 cycles. The resulting PCR product was gel-purified (2% agarose) with NucleoSpin® Gel and PCR Clean-up (Macherey–Nagel) and then cloned into the pGEMT Easy Vector System (Promega) following the manufacturers’ protocols. For all samples, 10 colonies were randomly chosen, and DNA was purified using NucleoSpin® 96 Plasmid (Macherey–Nagel) and sequenced with a 3730 DNA Analyzer (Applied Biosystems). Results were transformed into percentages of CpGs showing methylation.

### Gene expression analysis

For quantitative RT-PCR (qRT-PCR), 2 μg of total RNA was reverse-transcribed using the ThermoScript™ RT-PCR System (Invitrogen) according to the manufacturer's recommendations. Reactions for qRT-PCR were performed in triplicate on an Applied Biosystems 7,900HT Fast Real-Time PCR using 25–50 ng cDNA and TaqMan gene expression assays (LINC00473: Hs03677577_m1; GAPDH: Hs02758991_g1) as previously described [14]. GAPDH was used as an endogenous control, and water as a negative control. RNA-seq data of *LINC00473* from CRC cell lines and normal colon mucosa were obtained from the public database Gene Expression Omnibus (GEO) (GSE138734). RNA-seq data of *LINC00473* in CRC primary tumors and matched normal tissues were obtained from TCGA.

### DNA methylation analysis of 450K array data

To analyze the DNA methylation levels of the *LINC00473* promoter in CRC cell lines, we obtained 450K array data (β-values) from the public database GEO (GSE49143). For the methylation analysis of *LINC00473* promoter in primary colorectal tumors and matched normal tissues, we obtained the β-values of the 450K array from TCGA.

### Methylation analysis of the LINC00473 promoter in colorectal tissues by pyrosequencing

The methylation status of the *LINC00473* promoter was analyzed in primary colorectal tumors and matched normal tissues by pyrosequencing. Primers for PCR amplification and sequencing were designed using PyroMark Assay Design 2.0 software (Qiagen). 500 ng of DNA was bisulfite-converted with the EZ-96 DNA Methylation kit (Zymo Research) and used as a template for subsequent PCR. Before pyrosequencing, PCR products were observed on 2% agarose gels. Pyrosequencing and methylation quantification were performed in a PyroMark Q96 System (Qiagen) according to the manufacturer’s instructions. CpG site methylation was quantified using Pyro Q-CpG 1.0.9 (Qiagen). Water was used as negative control.

### Methylation analysis of the *LINC00473* promoter in cfDNA by qMSP

The methylation levels of the *LINC00473* promoter in plasma cfDNA were determined by quantitative methylation-specific PCR (qMSP) in a StepOne Plus system (Applied Biosystems). cfDNA (15–50 ng) was bisulfite-converted with the EZ DNA Methylation-Lightning Kit (Zymo Research) according to the manufacturer’s recommendations. Each reaction contained 2 µL of bisulfite-converted cfDNA as a template, 10 µl Power SYBR™ Green PCR Master Mix (Thermo Fisher) and 150 nM each forward and reverse primers in a total volume of 20 µl. Thermocycling conditions in the StepOne Plus system were as follows: 10 min at 95 °C, followed by 50 cycles of 94 °C for 15 s and 60 °C for 30 s. Water was included as a no-template control. HCT-116 and normal leukocytes (NLs) were used as positive controls for methylation and unmethylation, respectively. All the samples and controls were analyzed in triplicate. The DNA methylation level was expressed as a percentage of methylation (%) according to the following formula [30]: Methylation (%) = 100/[1 + 2^(CT_CG_–CT_TG_)], where CT_CG_ and CT_TG_ represent, respectively, the threshold cycle (CT) of the methylation and unmethylation status of the *LINC00473* promoter.

### Methylation analysis of the *LINC00473* promoter in cfDNA by ddPCR

Methylation of the *LINC00473* promoter was analyzed by droplet digital PCR (ddPCR) in a QX200 system (Bio-Rad). cfDNA (30–50 ng) was bisulfite-converted with the EZ DNA Methylation-Lightning Kit (Zymo Research) according to the manufacturer’s recommendations. A custom Bio-Rad assay to detect *LINC00473* methylation (*LINC00473*-M) or unmethylation (*LINC00473*-U) was designed. First, a multiplex preamplification reaction was performed using ~ 2 ng of bisulfite-converted DNA, 25 μl SsoAdvanced™ PreAmp Supermix (Bio-Rad), 0.5 μl of *LINC00473*-M and 0.5 μl of *LINC00473*-U in a total volume of 50 μl. PCR conditions were as follows: 3 min at 95 °C, 10 cycles of 95 °C for 15 s and 56.2 °C for 4 min, and a final hold step of 4 °C. Next, a multiplex reaction mix was prepared by combining 2 µL of the preamplification product, 11 μl ddPCR Supermix for Probes (No dUTP) (Bio-Rad), 2.2 μl of *LINC00473*-M and 2.2 μl of *LINC00473*-U in a total volume of 22 μl. The QX200™ Droplet Generator (Bio-Rad) was used to generate droplets. Thermocycling conditions were as follows: 10 min at 95 °C, 40 cycles of 95 °C for 15 s and 56.2 °C for 30 s; 98 °C for 10 min and a final hold step of 4 °C. The temperature ramp increment was 2.5 °C/s for all steps. Droplets were counted and analyzed using the QX200™ Droplet Reader (Bio-Rad), and QuantaSoft analysis (Bio-Rad) was performed to acquire data. Water was included as a no-template control, and HCT-116 and NL as positive controls for methylation and unmethylation, respectively. Reactions were performed in triplicate. DNA methylation was expressed according to the following formula: Methylation (%) = [*M*/(*U* + *M*)] × 100, where *M* represents the copies/μl of methylated cfDNA, and U the copies/μl of unmethylated cfDNA.

### Statistical analysis

The Kolmogorov–Smirnov test was used to evaluate the normality of the distribution of the data. Subsequently, the nonparametric Mann–Whitney *U* test was used for the comparison of methylation data. Nonparametric regression with generalized additive models (GAMs) [31] was used to evaluate the effect of age on methylation levels. To assess the diagnostic accuracy, a receiver operating characteristic (ROC) curve was generated. To obtain the greatest combination of sensitivity and specificity, the Youden index (J) was used: J = sensitivity + specificity − 1 [32]. The positive predictive value (PPV) and negative predictive value (NPV) were calculated: PPV = true positive/(true positive + false positive); NPV = true negative/(true negative + false negative). Correlation analysis between DNA methylation and expression levels was evaluated using Pearson’s coefficient. GraphPad Prism 7.0 software was used for statistical analysis and graphic representation. All expressed *p* values were calculated with two-tailed tests and were considered significant when the *p* value < 0.05.

## Supplementary Information

**Additional file 1: Figure S1.** Expression levels of *LINC00473* in colorectal cancer tissues. Expression levels of *LINC00473* were determined in tissues from primary colorectal cancer and matched normal colorectal mucosa (controls) by RNA-seq data obtained from The Cancer Genome Atlas (TCGA). P, *p* value analyzed by Mann–Whitney U test. Ctrl, controls; CRC, colorectal cancer.

**Additional file 2: Figure S2.** Evaluation of the diagnostic accuracy of the methylation of *LINC00473* to detect colorectal cancer stages. ROC curve analysis evaluating the methylation of *LINC00473* for the detection of CRC at stage I (A), II (B), III (C) and IV (D), in tissue samples from primary colorectal cancer and matched normal colorectal mucosa (controls) by 450K array (Cohort 1). P, *p*-value analyzed by ROC curve; AUC, area under the ROC curve; Ctrl, controls; CRC, colorectal cancer; Se, sensitivity; Sp, specificity.

**Additional file 3: Figure S3.** Validation of the diagnostic accuracy of the methylation of *LINC00473* to detect colorectal cancer stages. ROC curve analysis evaluating the methylation of *LINC00473* for the detection of CRC at stage I (A), II (B), III (C) and IV (D), in tissue samples from primary colorectal cancer and matched normal colorectal mucosa (controls) by pyrosequencing (Cohort 2). P, *p*-value analyzed by ROC curve; AUC, area under the ROC curve; Ctrl, controls; CRC, colorectal cancer; Se, sensitivity; Sp, specificity.

**Additional file 4: Figure S4.** Methylation levels of *LINC00473* in tissue colorectal polyps and colorectal cancer. **A-B** Comparison of methylation of LINC00473 between tissues from colorectal polyps, N-ACP, ACP and CRC and matched normal colorectal mucosa (controls) analyzed by pyrosequencing (Cohort 3). **C-D** ROC curve analysis evaluating the methylation of LINC00473 for the detection of colorectal polyps and colorectal cancer with respect to controls (Cohort 3). Horizontal lines represent mean methylation levels of *LINC00473*. P, *p*-value analyzed by Mann–Whitney U test or ROC curve; AUC, area under the ROC curve; Ctrl, controls; P, polyps; CRC, colorectal cancer; N-ACP, non-advanced colorectal polyps; ACP, advanced colorectal polyps.

**Additional file 5: Figure S5.** Validation of the methylation levels of *LINC00473* to detect tissue precancerous lesions. **A-C** Methylation levels of *LINC00473* promoter in tissues from premalignant colorectal polyps and normal colorectal mucosa (controls) by pyrosequencing (Cohort 4). **D-E** ROC curve analysis evaluating the methylation of *LINC00473* promoter for the detection of premalignant colorectal polyps (Cohort 4). Horizontal lines represent mean methylation levels of *LINC00473*. P, *p*-value analyzed by Mann–Whitney U test or ROC curve; AUC, area under the ROC curve; Ctrl, controls; P, polyps; N-ACP, non-advanced colorectal polyps; ACP, advanced colorectal polyps.

**Additional file 6: Figure S6.** Clinical utility of LINC00473 methylation for the noninvasive detection of colorectal cancer during the follow-up. Methylation levels of *LINC00473* promoter (blue color) were evaluated in serial plasma cfDNA samples at clinically relevant time points from 6 randomly selected metastatic CRC patients by ddPCR. CEA (red color) was also analyzed in the same patients. ddPCR, droplet digital PCR; CEA, carcinoembryonic antigen; CRC, colorectal cancer; 1L, first-line therapy initiation; PD, progressive disease; PR partial response; Pre-PTR, pre-primary tumor resection; Pre-MR, pre-metastases resection; SD, stable disease; F-U, follow-up

**Additional file 7. Table S1.** General description of the patient cohorts included in the study.

**Additional file 8. Table S2.** Demographic and clinical characteristics of the patient cohorts included in the study.

**Additional file 9. Table S3.** Demographic and clinical characteristics of metastatic colorectal cancer patients from cohort 8.

**Additional file 10.** Methods. Inclusion and exclusion criteria.

## Data Availability

The methylation and expression data obtained from the 450K array and RNA-seq used in this study are publicly available in TCGA and GEO (GSE49143 and GSE138734).

## Abbreviations

ACP: Advanced colorectal polyp
AN: Advanced neoplasia
AUC: Area under the curve
AZA: 5-Aza-2′-deoxycytidine
CpGI: CpG island
CRC: Colorectal cancer
*LINC00473*: Long intergenic non-protein coding RNA 473
N-ACP: Non-advanced colorectal polyp
ROC: Receiver operating characteristic
