# Supplementary figures and images for: Noninvasive early detection of colorectal cancer by hypermethylation of the LINC00473 promoter in plasma cell-free DNA
Source: Clin Epigenetics. 2022 Jul 9;14:86. doi: 10.1186/s13148-022-01302-x (PMC9271259; doi:10.1186/s13148-022-01302-x)

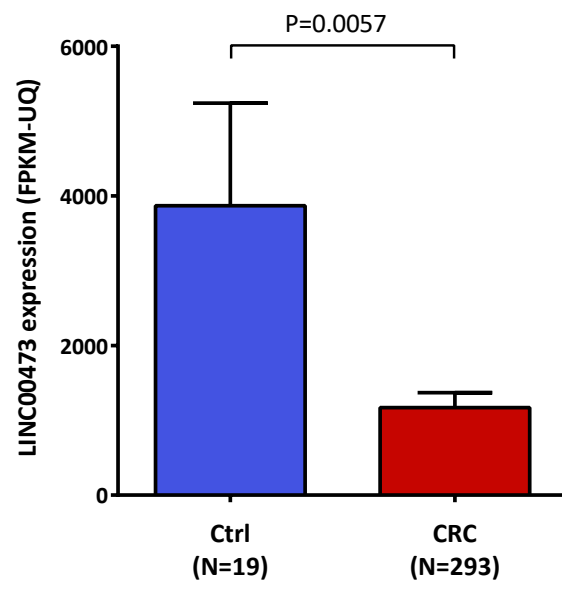

Supplement: Supplementary file 1 — Additional file 1: Figure S1. Expression levels of LINC00473 in colorectal cancer tissues. Expression levels of LINC00473 were determined in tissues from primary colorectal cancer and matched normal colorectal mucosa (controls) by RNA-seq data obtained from The Cancer Genome Atlas (TCGA). P, p value analyzed by Mann–Whitney U test. Ctrl, controls; CRC, colorectal cancer. [file 13148_2022_1302_MOESM1_ESM.pdf]

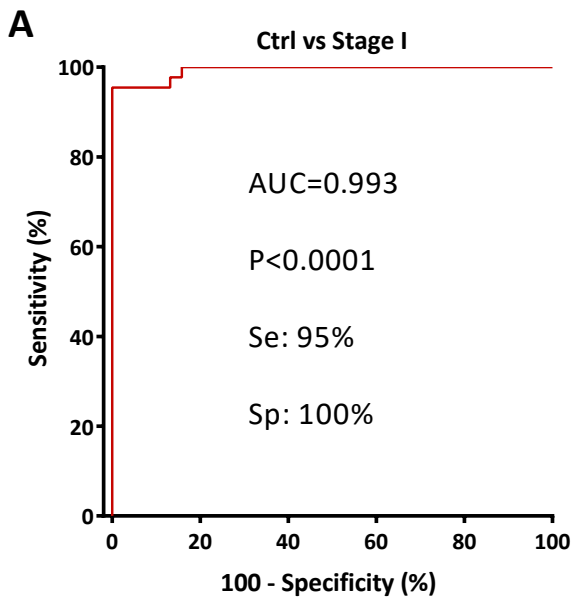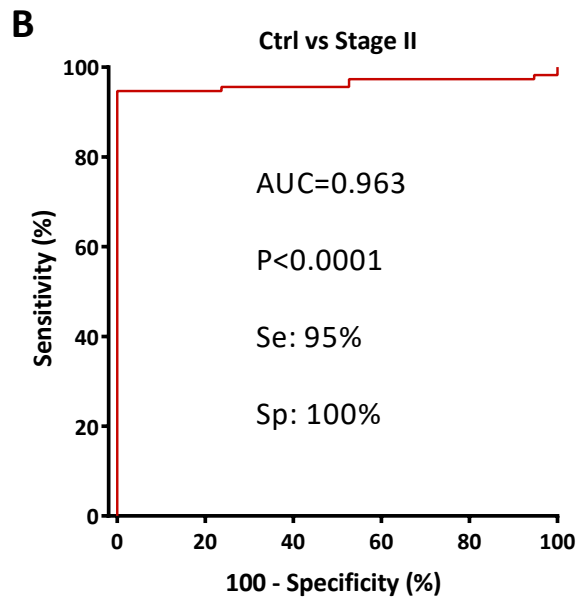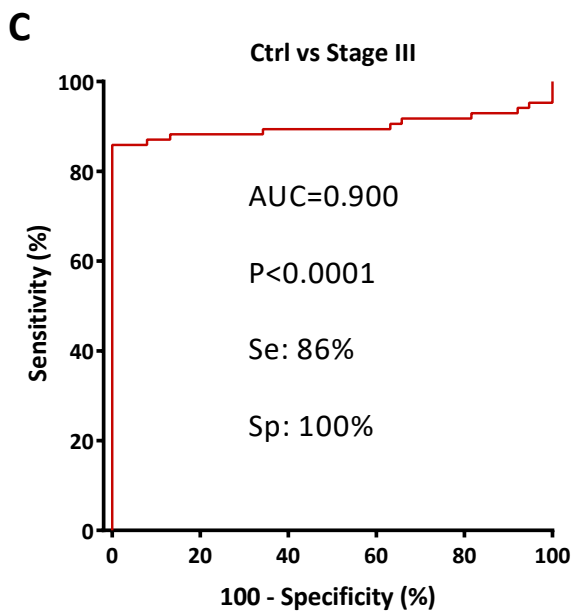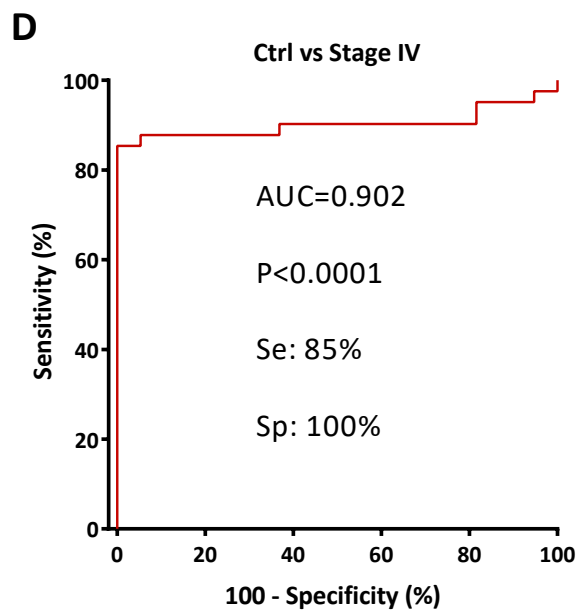

Supplement: Supplementary file 2 — Additional file 2: Figure S2. Evaluation of the diagnostic accuracy of the methylation of LINC00473 to detect colorectal cancer stages. ROC curve analysis evaluating the methylation of LINC00473 for the detection of CRC at stage I (A), II (B), III (C) and IV (D), in tissue samples from primary colorectal cancer and matched normal colorectal mucosa (controls) by 450K array (Cohort 1). P, p-value analyzed by ROC curve; AUC, area under the ROC curve; Ctrl, controls; CRC, colorectal cancer; Se, sensitivity; Sp, specificity. [file 13148_2022_1302_MOESM2_ESM.pdf]

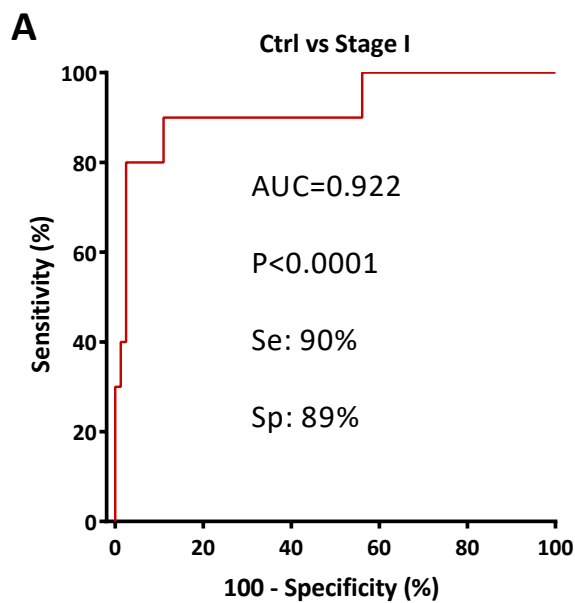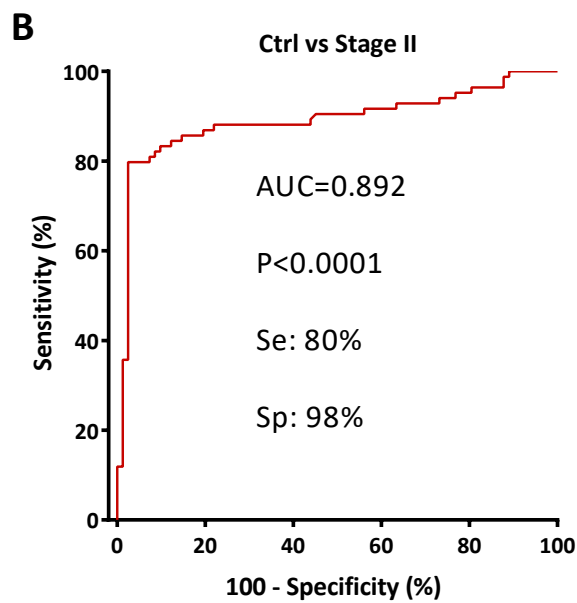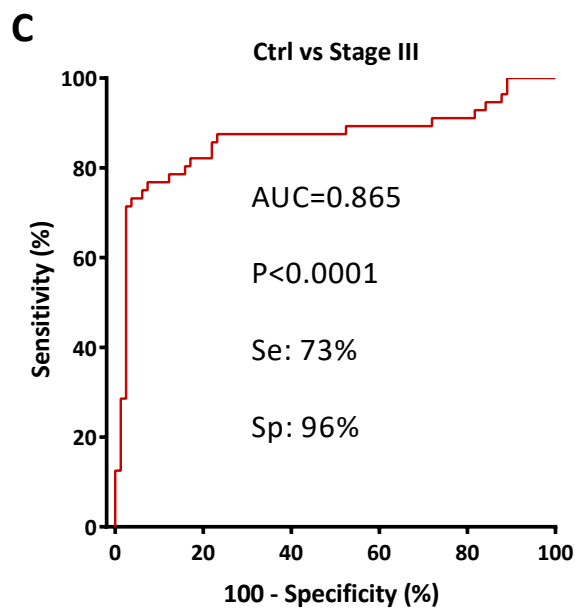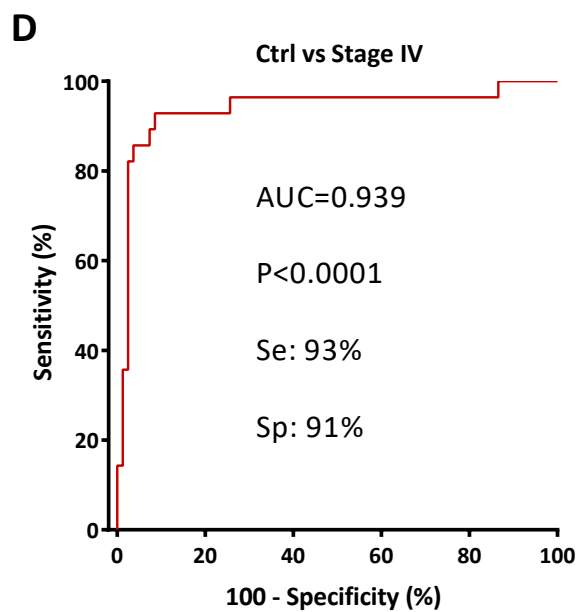

Supplement: Supplementary file 3 — Additional file 3: Figure S3. Validation of the diagnostic accuracy of the methylation of LINC00473 to detect colorectal cancer stages. ROC curve analysis evaluating the methylation of LINC00473 for the detection of CRC at stage I (A), II (B), III (C) and IV (D), in tissue samples from primary colorectal cancer and matched normal colorectal mucosa (controls) by pyrosequencing (Cohort 2). P, p-value analyzed by ROC curve; AUC, area under the ROC curve; Ctrl, controls; CRC, colorectal cancer; Se, sensitivity; Sp, specificity. [file 13148_2022_1302_MOESM3_ESM.pdf]

**A**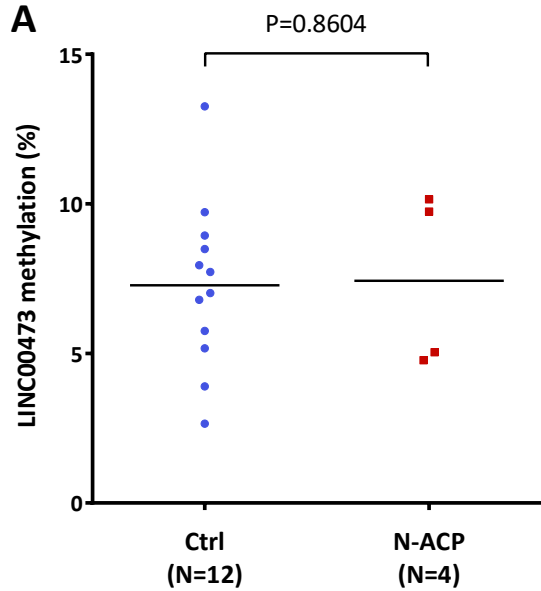**B**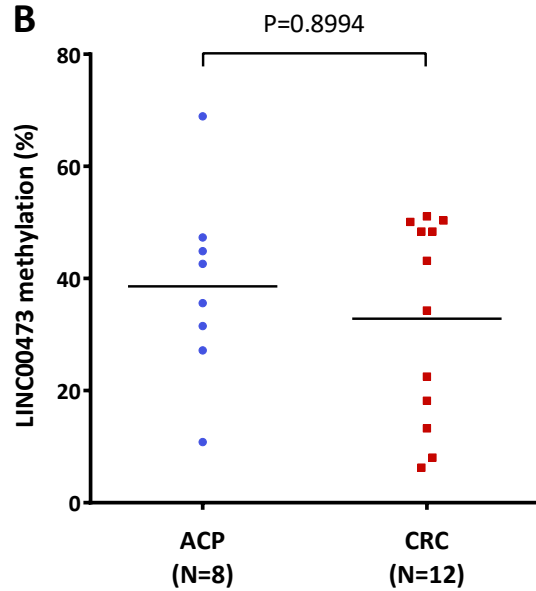**C**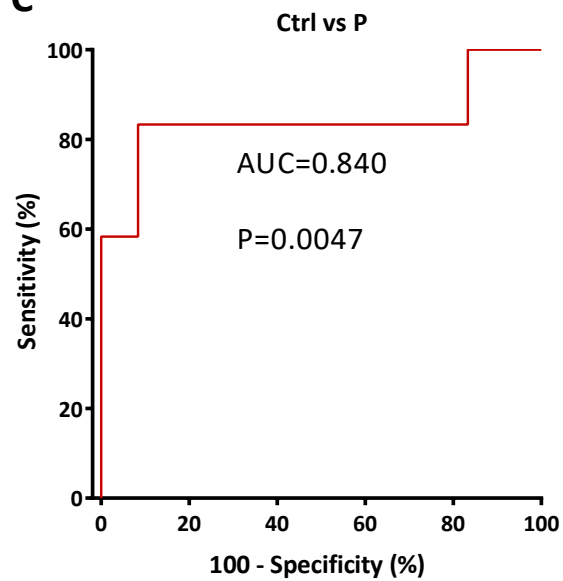**D**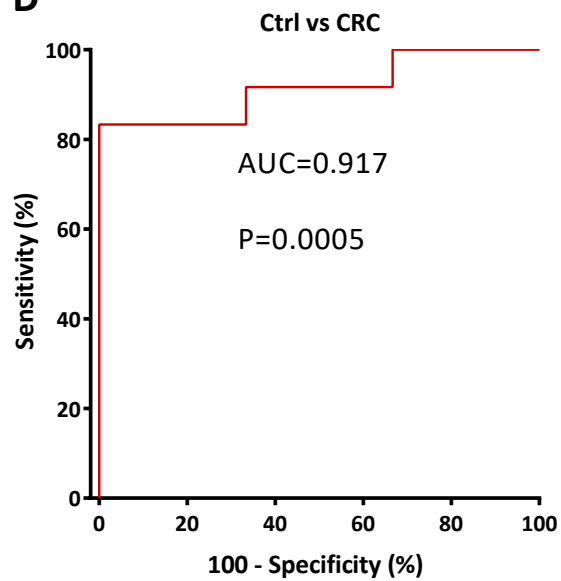

Supplement: Supplementary file 4 — Additional file 4: Figure S4. Methylation levels of LINC00473 in tissue colorectal polyps and colorectal cancer. A-B Comparison of methylation of LINC00473 between tissues from colorectal polyps, N-ACP, ACP and CRC and matched normal colorectal mucosa (controls) analyzed by pyrosequencing (Cohort 3). C-D ROC curve analysis evaluating the methylation of LINC00473 for the detection of colorectal polyps and colorectal cancer with respect to controls (Cohort 3). Horizontal lines represent mean methylation levels of LINC00473. P, p-value analyzed by Mann–Whitney U test or ROC curve; AUC, area under the ROC curve; Ctrl, controls; P, polyps; CRC, colorectal cancer; N-ACP, non-advanced colorectal polyps; ACP, advanced colorectal polyps. [file 13148_2022_1302_MOESM4_ESM.pdf]

**A**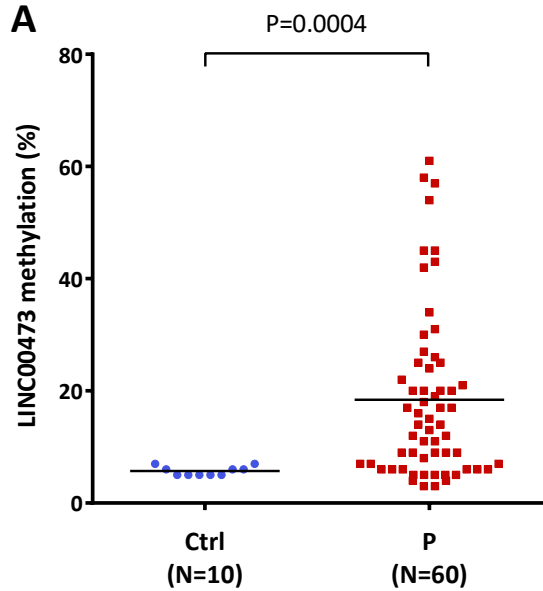**B**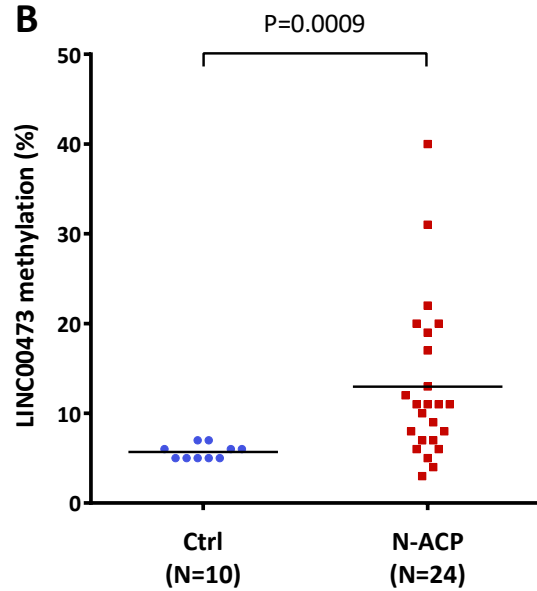**C**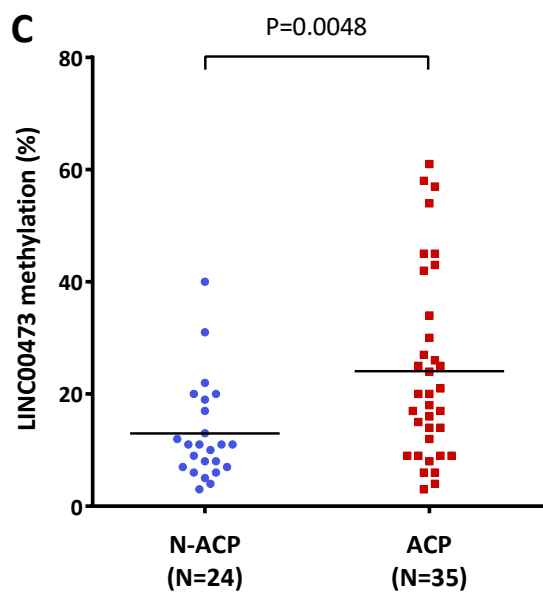**D**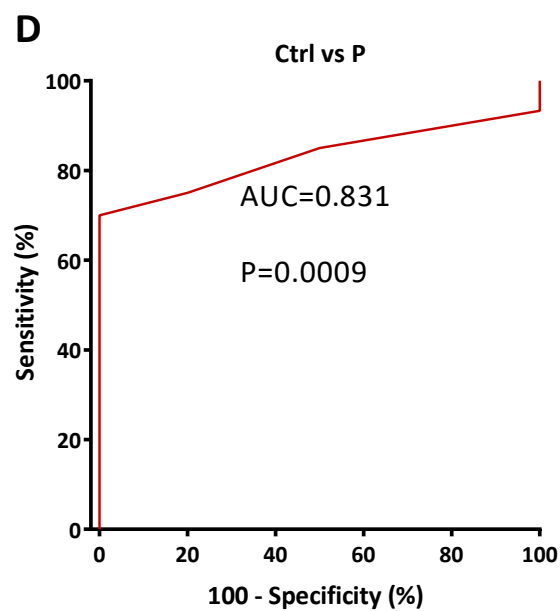**E**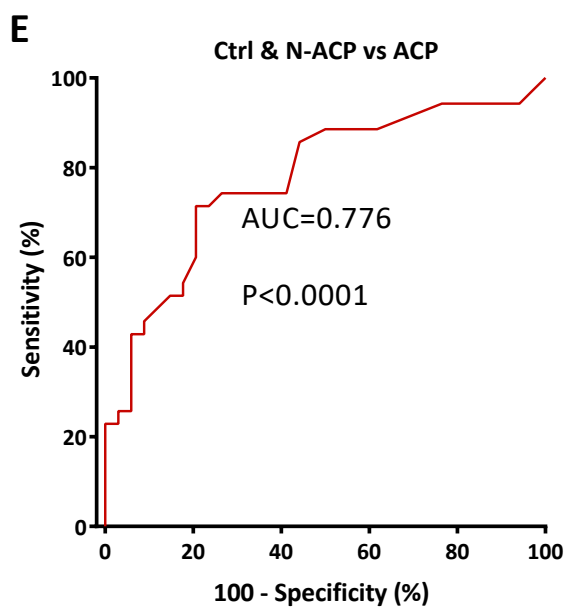

Supplement: Supplementary file 5 — Additional file 5: Figure S5. Validation of the methylation levels of LINC00473 to detect tissue precancerous lesions. A-C Methylation levels of LINC00473 promoter in tissues from premalignant colorectal polyps and normal colorectal mucosa (controls) by pyrosequencing (Cohort 4). D-E ROC curve analysis evaluating the methylation of LINC00473 promoter for the detection of premalignant colorectal polyps (Cohort 4). Horizontal lines represent mean methylation levels of LINC00473. P, p-value analyzed by Mann–Whitney U test or ROC curve; AUC, area under the ROC curve; Ctrl, controls; P, polyps; N-ACP, non-advanced colorectal polyps; ACP, advanced colorectal polyps. [file 13148_2022_1302_MOESM5_ESM.pdf]

**Case #1**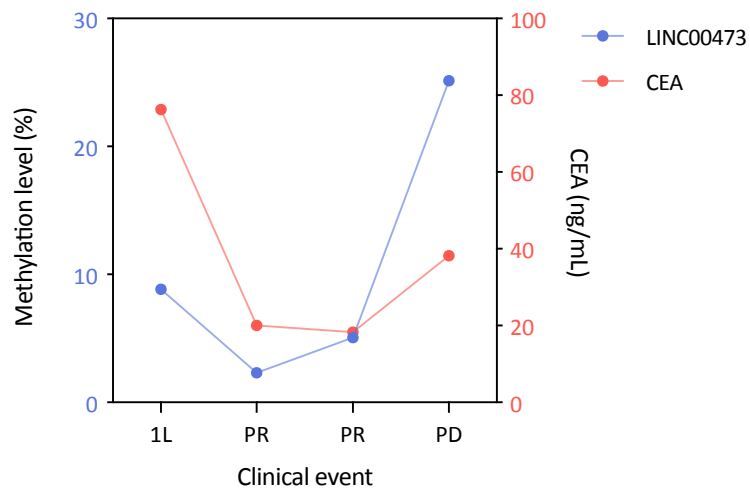**Case #2**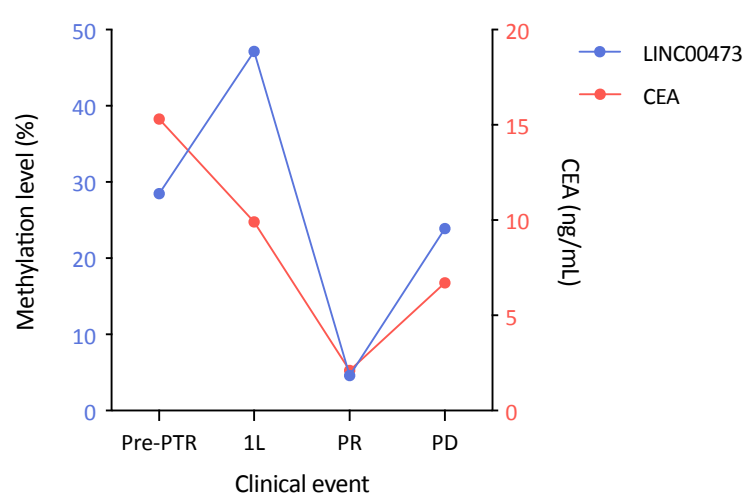**Case #3**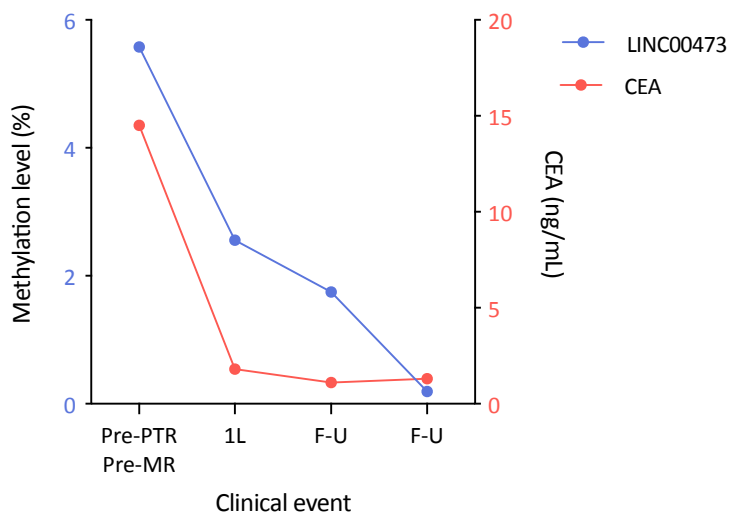**Case #4**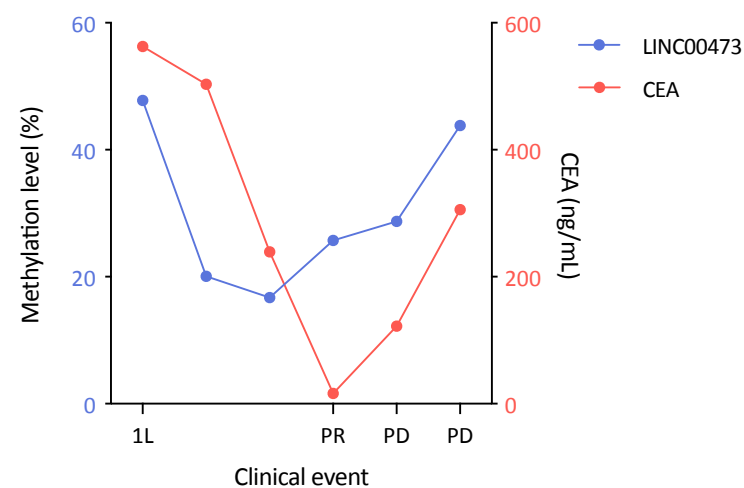**Case #5**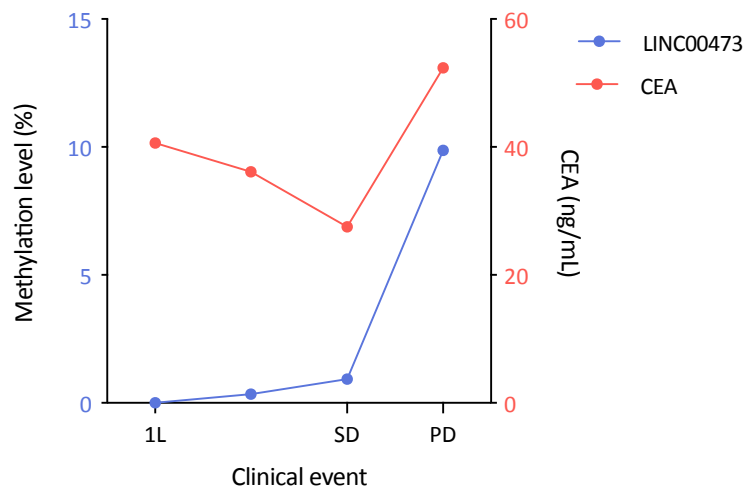**Case #6**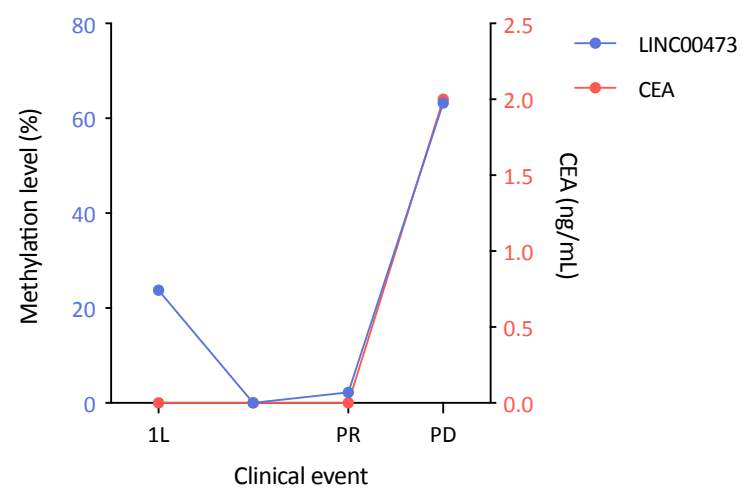

Supplement: Supplementary file 6 — Additional file 6: Figure S6. Clinical utility of LINC00473 methylation for the noninvasive detection of colorectal cancer during the follow-up. Methylation levels of LINC00473 promoter (blue color) were evaluated in serial plasma cfDNA samples at clinically relevant time points from 6 randomly selected metastatic CRC patients by ddPCR. CEA (red color) was also analyzed in the same patients. ddPCR, droplet digital PCR; CEA, carcinoembryonic antigen; CRC, colorectal cancer; 1L, first-line therapy initiation; PD, progressive disease; PR partial response; Pre-PTR, pre-primary tumor resection; Pre-MR, pre-metastases resection; SD, stable disease; F-U, follow-up [file 13148_2022_1302_MOESM6_ESM.pdf]
