# Supplementary material for: Noninvasive early detection of colorectal cancer by hypermethylation of the LINC00473 promoter in plasma cell-free DNA
Source: Clin Epigenetics. 2022 Jul 9;14:86. doi: 10.1186/s13148-022-01302-x (PMC9271259; doi:10.1186/s13148-022-01302-x)
Supplement: Supplementary file 7 — Additional file 7. Table S1. General description of the patient cohorts included in the study. [file 13148_2022_1302_MOESM7_ESM.pdf]

Supplementary Table S1. General description of the patient cohorts included in the study.

Cohort ID	Description	Institution/Hospital
1	Primary colorectal tumors and matched normal colorectal mucosa	The Cancer Genome Atlas (TCGA), NCBI, USA
2		Bellvitge Biomedical Research Institute (IDIBELL), Spain
3	Primary colorectal tumors and matched normal colorectal mucosa and colorectal polyps	Department of Molecular Diagnostics and Experimental Therapeutics (MDET) of the City of Hope National Medical Center, USA
4	Polyps and normal mucosa from non-cancer patients	Complejo Hospitalario Universitario de Ferrol, Spain
5	Plasma samples from self-declared healthy controls and CRC patients	Complejo Hospitalario Universitario de Compostela, Spain
6	Plasma samples from self-declared healthy controls and patients with ACPs presenting at least one polyp >10 mm previously confirmed by colonoscopy.	
7	Plasma samples obtained either prior to a scheduled colonoscopy as part of standard CRC screening or prior to colonic surgery for primary tumor	Complejo Hospitalario Universitario de Vigo, Instituto Valenciano de Oncología, Hospital Universitario de Burgos, Hospital General Universitario de Alicante, Complejo Hospitalario Universitario de Ourense and Hospital Clínico Universitario Lozano Blesa de Zaragoza, Spain.
8	Serial plasma samples collected at different clinically relevant time points from six randomly selected metastatic CRC (mCRC) patients under first-line standard chemotherapy	Complejo Hospitalario Universitario de Santiago de Compostela, Spain
