# Supplementary material for: Noninvasive early detection of colorectal cancer by hypermethylation of the LINC00473 promoter in plasma cell-free DNA
Source: Clin Epigenetics. 2022 Jul 9;14:86. doi: 10.1186/s13148-022-01302-x (PMC9271259; doi:10.1186/s13148-022-01302-x)
Supplement: Supplementary file 8 — Additional file 8. Table S2. Demographic and clinical characteristics of the patient cohorts included in the study. [file 13148_2022_1302_MOESM8_ESM.pdf]

Supplementary Table S2. Demographic and clinical characteristics of the patient cohorts included in the study.

	Colorectal tissue (FFPE)									Plasma (cfDNA)						
	Cohort 1 (N=331)		Cohort 2 (N=262)		Cohort 3 (N=36)			Cohort 4 (N=70)		Cohort 5 (N=54)		Cohort 6 (N=58)		Cohort 7 (N=57)		
**Characteristics**	Ctrl (N=38)	CRC (N=293)	Ctrl (N=82)	CRC (N=180)	Ctrl (N=12)	Polyps (N=12)	CRC (N=12)	Ctrl (N=10)	Polyps (N=60)	Ctrl (N=28)	CRC (N=26)	Ctrl (N=34)	ACP (N=24)	Ctrl (N=19)	ACP (N=17)	CRC (N=21)
**Mean age, years (range)**	69 (42-90)	65 (31-90)	66 (31-90)	67 (31-91)	71 (49-89)	66 (47-87)	64	64 (62-82)	65 (50-88)	51	67	29	69	53 (21-88)	66 (54-83)	71 (50-88)
**Gender**																
Male	21 (55%)	157 (54%)	46 (56%)	114 (63%)	7	11 (91.7%)	3 (25%)	6 (60%)	42 (70%)	12 (42.9%)	17 (65.4%)	17 (50.0%)	14 (58.3%)	6 (31.6%)	14 (82.4%)	11 (52.3%)
Female	17 (45%)	136 (46%)	36 (44%)	66 (37%)	5	1 (8.3%)	9 (75%)	4 (40%)	18 (30%)	16 (57.1%)	9 (34.6%)	17 (50.0%)	10 (41.7%)	13 (68.4%)	3 (17.6%)	9 (42.9%)
Unknown	-	-	-	-	-	-	-	-	-	-	-	-	-	-	-	1 (4.8)
**CRC Stage**																
I	-	44 (15.0%)	-	10 (5.5%)	-	-	7 (58.3%)	-	-	-	-	-	-	-	-	2 (9.5%)
II	-	113 (38.6%)	-	84 (46.7%)	-	-	2 (16.7%)	-	-	-	-	-	-	-	-	2 (9.5%)
III	-	85 (29.0%)	-	56 (31.1%)	-	-	-	-	-	-	4 (15.4%)	-	-	-	-	3 (14.3%)
IV	-	41 (14.0%)	-	28 (15.6%)	-	-	3 (25%)	-	-	-	22 (84.6%)	-	-	-	-	8 (38.1%)
Unknown	-	10 (3.4%)	-	2 (1.1%)	-	-	-	-	-	-	-	-	-	-	-	6 (28.6%)
**Type of polyp**																
Hyperplastic	-	-	-	-	-	-	-	-	10 (16.7%)	-	-	-	-	-	-	-
Adenomatous	-	-	-	-	-	11 (91.7%)	-	-	20 (33.3%)	-	-	-	23 (95.8%)	-	17 (100%)	-
Serrated	-	-	-	-	-	1 (8.3%)	-	-	30 (50%)	-	-	-	1 (4.2%)	-	0 (0%)	-
Unknown	-	-	-	-	-	-	-	-	-	-	-	-	-	-	-	-
**Polyp status**																
N-ACP	-	-	-	-	-	4 (33.3%)	-	-	24 (40%)	-	-	-	-	-	-	-
ACP	-	-	-	-	-	8 (66.7%)	-	-	35 (58.3%)	-	-	-	24 (100%)	-	17 (100%)	-
Unknown	-	-	-	-	-	-	-	-	1 (1.7%)	-	-	-	-	-	-	-

Ctrl, control; CRC, colorectal, N-ACP, non-advanced colorectal polyp; ACP, advanced colorectal polyp.
