# Supplementary material for: Noninvasive early detection of colorectal cancer by hypermethylation of the LINC00473 promoter in plasma cell-free DNA
Source: Clin Epigenetics. 2022 Jul 9;14:86. doi: 10.1186/s13148-022-01302-x (PMC9271259; doi:10.1186/s13148-022-01302-x)
Supplement: Supplementary file 9 — Additional file 9. Table S3. Demographic and clinical characteristics of metastatic colorectal cancer patients from cohort 8. [file 13148_2022_1302_MOESM9_ESM.pdf]

Supplementary Table S3. Demographic and clinical characteristics of metastatic colorectal cancer patients from cohort 8.

Characteristics	Patients
**Size - no.**	6
**Age - years**	
Median (range)	61 (47- 81)
**Sex - no. (%)**	
Female	3 (50)
Male	3 (50)
**ECOG-PS - no. (%)**	
1	5 (83)
2	1 (17)
**Primary tumour site - no. (%)**	
Right colon	1 (17)
Transverse colon (proximal two thirds)	1 (17)
Left colon	5 (83)
Sigmoid colon	2 (33)
Rectosigmoid junction	2 (33)
Rectum	1 (17)
**RAS status - no. (%)**	
Mutated	3 (50)
KRAS mutation	3 (50)
No mutated	3 (50)
**BRAF status - no. (%)**	
No mutated	6 (100)
**MMR status - no. (%)**	
Preserved	5 (83)
Deficient	1 (17)
MLH1/PMS2 deficient	1 (17)
**TNM stage at diagnosis - no. (%)**	
I	1 (17)
IV	5 (83)
**Site of metastases - no. (%)**	
Liver	5 (83)
Lung	3 (50)
Peritoneum	1 (17)
**Number of metastatic sites - no. (%)**	
1	3 (50)
2	3 (50)
**Primary tumor resection - no. (%)**	
Yes	4 (67)
No	2 (33)
**CEA - no. (%)**	
≤5 ng/mL	1 (17)
>5 ng/mL	5 (83)
**Chemotherapy regimen - no. (%)**	
mFOLFOX6	4 (67)
FOLFIRI	1 (17)
5-FU/LV	1 (17)
**Antibody - no. (%)**	3 (50)
Anti-EGFR	3 (50)
Cetuximab	2 (33)
Panitumumab	1 (17)
