# Supplementary material for: Noninvasive early detection of colorectal cancer by hypermethylation of the LINC00473 promoter in plasma cell-free DNA
Source: Clin Epigenetics. 2022 Jul 9;14:86. doi: 10.1186/s13148-022-01302-x (PMC9271259; doi:10.1186/s13148-022-01302-x)
Supplement: Supplementary file 10 — Additional file 10. Methods. Inclusion and exclusion criteria. [file 13148_2022_1302_MOESM10_ESM.pdf]

Supplementary Methods

Inclusion and exclusion criteria

Tissue cohorts:

Inclusion criteria are those already described in the Description section of Supplementary Table S1. Exclusion criteria included samples from patients who have previously received chemotherapy or radiotherapy.

Plasma cohorts:

Cohort 5 and cohort 6: Inclusion criteria included either subjects self-declared healthy (without confirmatory colonoscopy), or subjects histologically diagnosed of advanced colorectal polyps (ACPs) who were referred to colonoscopy in the context of a colorectal cancer (CRC) screening program, or subjects diagnosed with CRC scheduled for surgery.

Cohort 7: Inclusion criteria included subjects who were referred to colonoscopy in the context of a CRC screening program and after colonoscopy they have been classified as healthy controls, or CRC or ACP based on histological diagnosis. In the case of CRC, subjects histologically diagnosed with CRC scheduled for surgery were also permitted.

Commom exclusion criteria for cohorts 5 to 7 included patients (or controls) who have developed any another type of cancer in the previous 5 years, except curatively treated basal or squamous cell skin cancer, prostate intraepithelial neoplasm, carcinoma in-situ of the cervix, Bowen’s disease or very low/low risk prostate cancer; those who have previously received chemotherapy or radiotherapy; subjects previously diagnosed with any hereditary polyposis syndrome, Lynch syndrome, inflammatory bowel disease, or those with a first degree relative previously diagnosed with CRC.

Cohort 8: Inclusion criteria are those already described in the Description section of Supplementary Table S1. Exclusion criteria included samples from patients who have previously received chemotherapy or radiotherapy in the advanced CRC setting, and those who have developed any another type of cancer in the previous 5 years, except curatively treated basal or squamous cell skin cancer, prostate intraepithelial neoplasia, carcinoma in-situ of the cervix, Bowen's disease or very low/low risk prostate cancer. None of the 6 cases included in cohort 8 were diagnosed with any hereditary polyposis syndrome, Lynch syndrome, or inflammatory bowel disease.
